# Community input in a genomic health implementation program: Perspectives of a community advisory group

**DOI:** 10.3389/fgene.2022.892475

**Published:** 2022-07-22

**Authors:** Miranda E. Vidgen, Katrina Cutler, Jessica Bean, David Bunker, Lindsay F. Fowles, Louise Healy, Gary Hondow, Satrio Nindyo Istiko, Aideen M. McInerney-Leo, Gregory Pratt, Deborah Robins, Nicola Waddell, Erin Evans

**Affiliations:** ^1^ QIMR Berghofer Medical Research Institute, Herston, QLD, Australia; ^2^ Queensland Genomics, Herston, QLD, Australia; ^3^ Health Translation Queensland, Herston, QLD, Australia; ^4^ Queensland Genomics Community Advisory Group, Brisbane, QLD, Australia; ^5^ Genetic Health Queensland, Royal Brisbane and Women’s Hospital, Herston, QLD, Australia; ^6^ Dermatology Research Centre, University of Queensland Diamantina Institute, University of Queensland, Brisbane, QLD, Australia; ^7^ Health Consumers Queensland, Brisbane, QLD, Australia

**Keywords:** community and consumer engagement, community advisory boards, genomic, genetics, emerging technologies, health implementation science, public participation, community and consumer involvement

## Abstract

Consumer and community engagement (CCE) in the implementation of genomics into health services and associated research is needed to ensure that changes benefit the affected patients. Queensland Genomics was a program to implement genomics into a public health service. We describe its Community Advisory Group’s (CAG) structure and function and provide recommendations based on the CAG members’ perspectives. The CAG provided advice to the Queensland Genomics program and its projects in an advisory capacity. The CAG was also resourced to develop and lead community-focused activities. Key enablers for CAG included; diversity of CAG members’ skills and experience, adequate resourcing, and the CAG’s ability to self-determine their direction. The CAG experienced limitations due to a lack of mechanisms to implement CCE in the Program’s projects. Here, we provide insights and commentary on this CAG, which will be useful for other initiatives seeking to undertake CCE in genomic research and health care.

## 1 Introduction

Consumer and community engagement (CCE) in research and health care is working in partnership with end-users to improve processes and outcomes (Consumer Health Forum of Australia, 2016) and address the ethical premise that consumers have a right to provide input when issues affect them (Sarrami-Foroushani et al., 2014). For a discipline like genomics, which has extensive personal, familial, and social implications, there is a distinct need for CCE in research and health service implementation (Wale et al., 2021).

In general, a Community Advisory Group (CAG) consists of community members working with organisation representatives to provide community and consumer perspectives (McClean and Trigger, 2017; Stewart et al., 2019). In health and medical services and research, CAGs have been; involved in individual research projects (Synnot et al., 2018; Mlambo et al., 2019), provided overarching advice at the institutional or organisational level (Gunatillake et al., 2020), and guided improvements to health service delivery (McClean and Trigger, 2017). The purpose, structure and responsibilities of CAGs vary considerably based on the rationale for their implementation. CAG responsibilities can include, but are not limited to; representing and advocating community perspectives, reviewing documents (for example, policies, study protocols, grants, and ethics), establishing a link between community and organisational representatives, and dissemination and collection of information within CAG members’ networks (Mlambo et al., 2019; Gunatillake et al., 2020). The variable purpose for individual CAGs means there is no singular framework or model to suit all settings. As such, there is study, program or service-specific implementation (Domecq et al., 2014).

Here we describe a CAG established to provide CCE within a program implementing genomics across the whole health system. We detail the CAG’s structure, which had the dual role of an advisory committee and developing community-led activities and research within the health implementation program. Finally, we provide recommendations for implementers based on CAG members’ experience as they move beyond an advisory capacity to activity-based leadership.

## 2 Program context

Queensland Genomics (“the Program”) was a 5-years program (2016–2021) that aimed to implement genomics into Queensland’s health care system via a series of health implementation initiatives and research projects (‘the Program’s projects’) that focused on whole of system capacity and capability building (Vidgen et al., 2021).

## 3 Community advisory group structure

### 3.1 Line of reporting

The CAG was established as an advisory group that reported to the Program’s Governance Board via the Executive Director, who attended CAG meetings as an ex-officio member (Supplementary Figure S1). The Program implemented the CAG as a mechanism to; incorporate patient and community views on planning and policy development, provide input on project design, advise on opportunities for patient and community engagement, and advocate on behalf of the community.

### 3.2 CAG members

**FIGURE 1:**
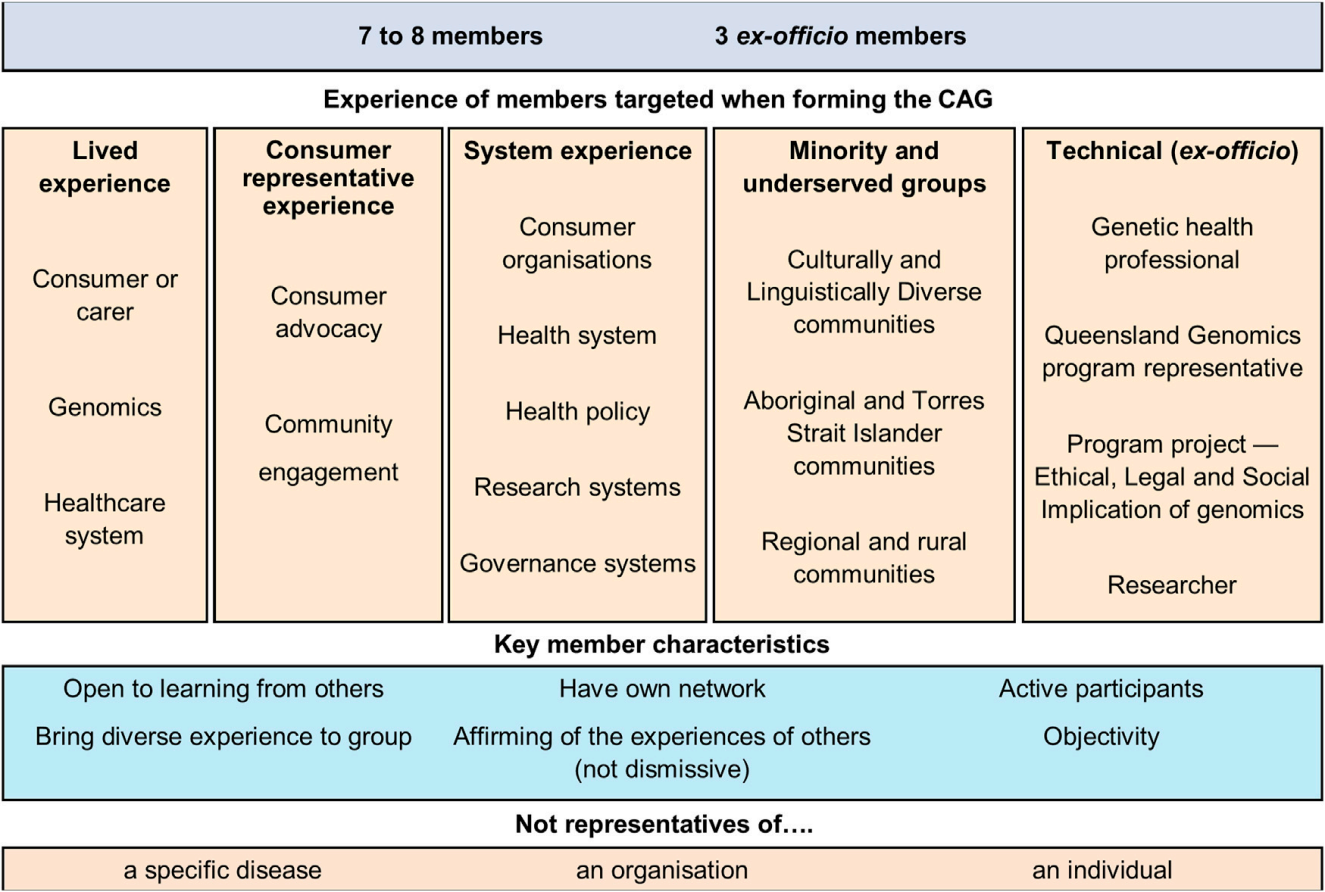
Community Advisory Group members’ characteristics and experience.

The selection of CAG members was a deliberate process by the Queensland Genomics business team and existing CAG members. Recruitment occurred through multiple methods; nomination of a person by key stakeholders, identification of suitable people at stakeholder meetings and events, and through expression of interest–circulated on behalf of the Program by health consumer organisations (for consumer recruitment) and health services (for genetic health professional recruitment). Applicants were considered based on targeted criteria, with a specific effort to recruit representatives with a broad range of experience (Figure 1).

The selected CAG members had diverse personal and professional backgrounds and skills - contributing specialist knowledge and expertise to the Program. They provided individual and system-level input from the perspective of consumers, carers, health delivery workers, medical researchers, consumer advocates, and community representatives (Figure 1). Members were not advocates for a particular organisation, disease area, or personal agenda. Members also provided perspectives from culturally and linguistically diverse communities, Aboriginal and Torres Strait Islander communities, and rural and regional communities. The CAG was established with the intent to be a network of networks. Therefore, all members came to the group with a diverse network of professional and community contacts.

The CAG ranged between ten to eleven members throughout the Program, with an annual renewal unless the member chose to withdraw. Most members continued for the Program’s duration, but some did take a leave of absence and return to the CAG to facilitate their health needs or carer responsibilities. Members were eligible to receive an honorarium to help support participation unless they were an investigator on a Program’s project, a Queensland Health employee, or a paid employee of a consumer, patient, carer or health peak body. The honorarium was based on local health advocacy policy (Health Consumers Queensland, 2015) and equated to 2.5 times the national minimum wage, with annual wage growth adjustments. Some members declined the honorarium payment for personal reasons.

The minimum participation for CAG members was attending 3-h quarterly meeting with completion of the associated reading. Participation in all other Program events or CAG-led activities was at the discretion of the individual member. CAG members were also remunerated when invited to attend Program events or meetings. However, all CAG members provided significantly more time to the Program than then minimum, based on their own availability and areas interests.

### 3.3 CAG strategy

The Program established the CAG with a broad Terms of Reference outlining scope and structure while ensuring the CAG had sufficient autonomy to set their direction and agenda. Shortly after establishment, the CAG developed its strategy to inform the group’s direction within the scope outlined by the Terms of Reference. The CAG strategy was developed through a workshop with all members and facilitated by the CAG Chair and iterative feedback from CAG members on the draft. The resulting CAG Strategy outlined the principles underpinning the direction of the CAG’s activities for the Program’s duration and included the CAG vision, objectives, themes of work, and action areas. Themes for the CAG focused on collaboration, equity and access, education, advocacy, foundation and prioritisation, and real-world context (Supplementary Figure S2).

### 3.4 Support for CAG

The Program allocated funds and resources for running the CAG. The CAG received administrative, logistical, and operational support from the Program’s Communication and Engagement Manager, who operated as the CAG Project Manager (Supplementary Figure S1). The Project Manager role included secretariat duties, e.g., preparation of meeting agendas with the Chair, organising working groups, recruitment of CAG members, undertaking actions identified in meetings, arranging travel for in-person meetings and arranging meetings with internal and external stakeholders (Supplementary Figure S1). The Program allocated additional business team staff when required. This additional support included; event planning and management, communications content development, and contracting and managing external suppliers.

## 4 CAG activities

### 4.1 CAG advisory activities for the program

**FIGURE 2:**
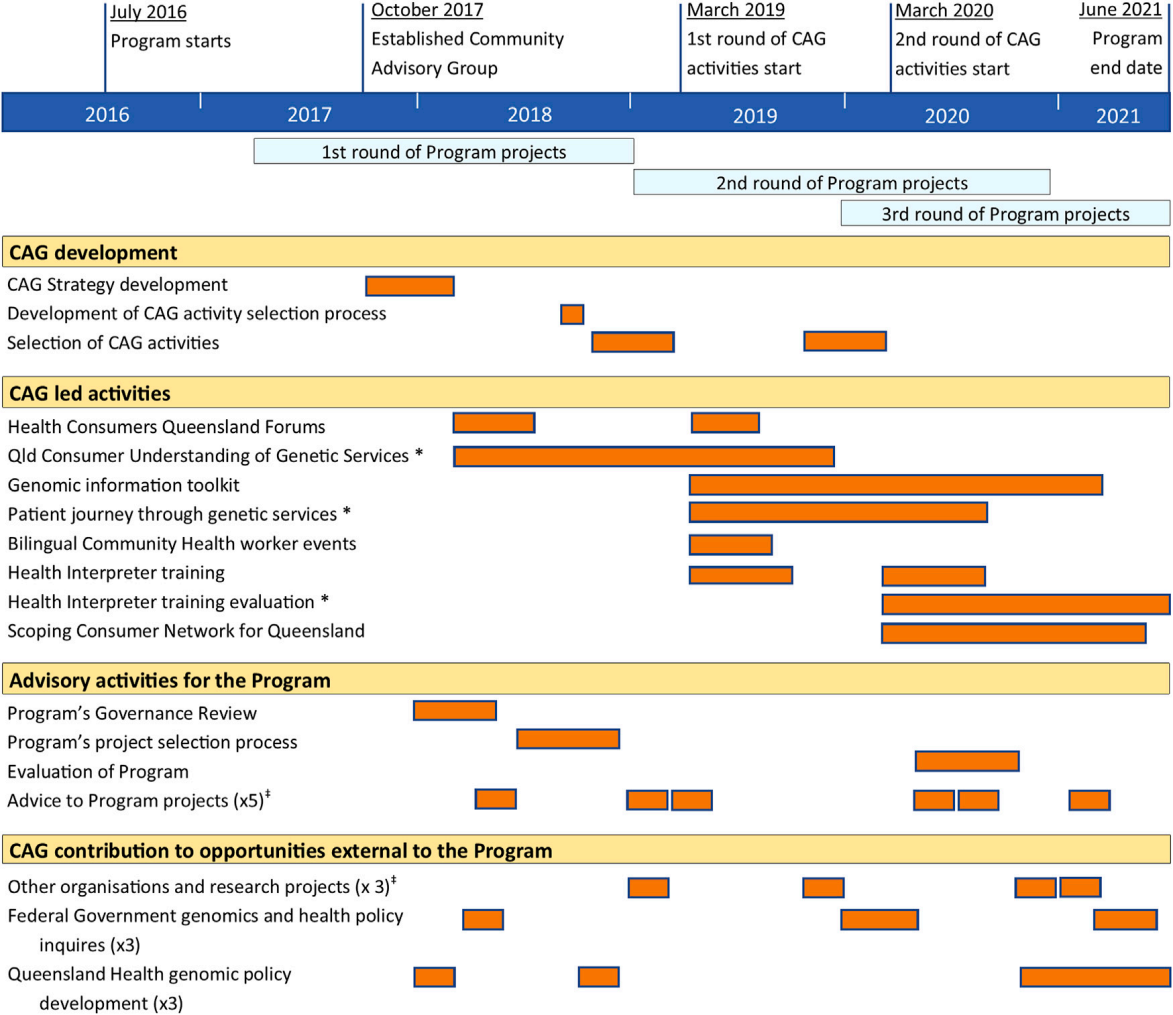
Summary and timeline of Community Advisory Group activities. * Research activities resulting in publication (Wallingford et al., 2020; Vidgen et al., 2022; Wallingford et al., 2022). ^‡^ Some projects were consulted multiple times.

Throughout the Program, the CAG undertook tasks nominated by the Program, including contribution to the Program Governance Review and the Program’s project selection processes (Figure 2). In addition, CAG members offered their perspective through advice or reviewing materials to the Program’s projects.

### 4.2 CAG-led activities

In the Program’s early phase, the CAG self-nominated areas of interest and associated activities aligned with the CAG Strategy. These activities came from CAG members’ networks and inquiries directly to the Program, including Federal Government Parliamentary health inquiries, other organisation and research projects, and Queensland Health policy planning for genomics (Figure 2).

During the Program’s later phases, the CAG identified, designed and ran research and non-research activities funded by the Program (Figure 2). The expectations were that the activities reflect areas of need for consumers and the community, which were not already addressed by the Program (Vidgen et al., 2021). There was an additional separate allocation of funds for the CAG to undertake activities in the later stages of the Program, which aligned with their strategic foci. The allocation of funds was critical to enable CAG members to take on leadership roles in developing and directing the activities while allocating day-to-day workload to paid staff and external contractors. These funds enabled the CAG to secure project officers and research assistants, contract specialist services (i.e., graphic designers, translators, facilitators), and costs associated with events.

CAG members nominated the CAG activities through a written expression of interest process. Members presented their ideas for nominated activities at a CAG meeting, with other members contributing their perspectives to develop the activities further. All CAG members nominated their preferred activities from the ideas presented and made recommendations to the Program’s Executive Director, who made the final selection. The Program’s selection was predominantly based on each activity’s viability within resource and time constraints.

For each CAG activity selected, the CAG formed working groups consisting of self-nominated CAG members (Supplementary Figure S1). These groups provided direction and oversight of the activity and provided advice on behalf of the broader CAG. Each activity reflected one or more “themes of work” outlined in the CAG’s strategic plan (Supplementary Figure S2). All CAG activities collaborated with external stakeholders (Supplementary Figure S1) that varied in style and scope depending on the activity’s needs.

Community education was achieved through presentations and seminars at Health Consumers Queensland Forums in 2018 and 2019. These presentations centred on integrating genomics into health care (2018) and facilitated a question and answer session to address community questions around genomics (2019). The community perspective at these forums was explored through surveys. These surveys were used to improve our knowledge of Queensland Consumers’ understanding of Genetic Services (Wallingford et al., 2020).

Multiple CAG activities focused on equity and access to health services and education. The CAG developed a series of written resources to help patients navigate the Queensland’s health system genomic services (known as the Genomics Information Toolkit). These patient resources were translated into five languages other than English: Arabic, Persian (Farsi), Vietnamese, Japanese and Chinese (simplified) (Queensland Genomics, 2021). The release of these resources was also supported by a targeted promotional campaign on social media. The Genomics Literacy in Multicultural Queensland projects provided information on genetics in the health system to people working with culturally and linguistically diverse communities. Forty Bilingual Community Health Workers attended a genetics in the health system education event. While ‘The Language of Genetics & Genomics: Healthcare Interpreter Training’ was developed and attended by 171 Health Interpreters over five sessions in 2019 and 2020 and was subsequently evaluated for effectiveness (Vidgen et al., 2022).

Advocacy, foundation and prioritisation, and real-world context themes (Supplementary Figure S2) were addressed by an activity mapping the patient journey through clinical genetic services (Wallingford et al., 2022) and scoping Queensland’s ongoing needs for a genomics consumer support network and health service advisory group. The scoping project was achieved through extensive community and stakeholder consultation. A report with recommendations from the scoping project was created and provided to Queensland Health’s Genomics Executive Working Group. This working group is developing a policy and strategy for genomics in Queensland’s health system after completing the Program, which includes a Person-Centred Care Expert Advisory Group and associated activities.

## 5 Evolution and impact of the CAG

Supporting the CAG to undertake community-led activities was a unique feature of this Program. This positioned CAG members as experts collaborating to identify and provide solutions to areas of unmet need rather than advisors to already established research projects or health service initiatives. This recognition of expertise enabled members to use their collective skills and knowledge to develop real-world outputs for consumers and the community. It provided opportunities for skills and knowledge development, as CAG members gained exposure to insights from other members and the activities of project officers and contractors.

The CAG was not established with the capacity to undertake multi-year, community-led activities. The Program’s trust in the CAG to undertake more substantial work came through the CAG’s demonstration of capability and readiness over their first 12-months (Figure 2) by developing and executing the CAG strategic plan and completing tasks nominated by the Program.

The efforts to establish the CAG’s readiness for community-led activities resulted in the secondary effect of improving their advisory capacity within the Program. The Program’s trust and relationship enabled the CAG to develop a voice that had influence. The CAG identified that the Program’s operation had insufficient mechanisms and opportunities for the CAG to provide CCE support to projects. The issue was raised by the CAG members several times throughout the Program. These discussions resulted in changes, including; the CAG being involved in the Program’s project selection process and the inclusion of new CCE requirements around expectations and reporting mechanisms for projects.

## 6 Discussion

### 6.1 Limitations experienced

Despite the Program’s desire to have embedded CCE across projects, the CAG was established concurrently with the Program’s first round of projects. So it was unable to contribute to this initial series of projects. While advocacy by the CAG for greater involvement in the Program and its projects resulted in improved CCE, the initial lack of mechanisms for CCE and the difficulty with the later implementation of CCE measures is evident in the fragmented engagement with the CAG and Program’s projects. Only 2 of the 15 clinical projects and 3 of 28 capability projects took up the CAG offer for consumer input. Some of the Program’s projects did incorporate CCE through direct recruitment of or consultation with disease-specific consumer or consumer organisation representatives rather than engaging with the CAG.

This inconsistent engagement stemmed from two areas. Outside of the Program, at the organisational level, there was a lack of support for CAG being involved in Program decision making, for example, being represented in the Program’s Board. The Program also experienced push-back from some project leaders who questioned the need for CCE in their projects. These experiences reflect two known barriers to effective implementation of CCE practices; implementers’ knowledge and experience, and the level of organisational support (Miller et al., 2017).

An area of need for organisation-wide implementation of CCE going forward, which was not captured by this Program, is the education of the implementers of CCE (i.e. clinical staff, project and initiative managers, and researchers). CAGs are key knowledge holders and advocates for developing and delivering educational interventions for CCE implementers by presenting examples and conveying their experiences and advice.

To have acceptance of CAGs, there needs to be cross-organisational normalisation of consumer and community involvement in health services and research (Chew-Graham, 2016; Miller et al., 2017; Pagatpatan and Ward, 2017). In this case, the evidence of the CAGs impact on the Program scope of activities has had a positive impact at the organisational level. As the health department has been planning for the post-program continuation of genomics implementation, person-centred healthcare has been identified as one of the strategic priority areas. This includes incorporating consumer and community perspectives.

### 6.2 Recommendations for CAGs and researchers

Based on the CAG member experiences, both positive and negative, a series of recommendations for CAGs and organisations implementing CAG as part of research or healthcare delivery are summarised in Supplementary Table S1. Here we explore three of the key recommendations identified by CAG members in further detail.

#### 6.2.1 CAG self-determination

CAGs formed by researchers, organisations, or health services often have specific tasks or ask to contribute in ways that reflect the organiser’s intent (Miller et al., 2017). While this CAG was not independent of the Program, it was supported to self-determine its objectives and operation, which was essential to how the CAG functioned. This autonomy was evident in the CAG’s ability to form and execute a strategic plan, manage CAG meeting and work priorities, and initiate communication with Program’s projects to promote the CAG’s CCE services.

The CAG’s self-determination of its activities significantly impacted the Program, the individual CAG members and the group as a collective. Primarily, these CAG activities facilitated meaningful dialogue with the Program on the needs of consumers and the community as a whole. It created awareness of issues and provided a mechanism for the Program to address unmet needs, using the CAG as an expert resource that defined and led activities. The ability to self-determine activities was beneficial to the CAG members’ motivation and engagement in the Program. It provided an opportunity for the CAG to contribute to the Program actively and gave the CAG responsibilities and ownership of self-determined activities.

#### 6.2.2 Experience and diversity of CAG members

There is debate over the level of experience CAG participants should have when recruited (Saunders et al., 2007; Synnot et al., 2018; Ehrlich et al., 2020). Engaging with consumers and community members with no experience in CCE or CAGs is thought to mitigate bias and improve equity of participation (Synnot et al., 2018). In this CAG, the recruitment of experienced and diverse members was viewed as a critical enabler. The CAG membership included experienced consumer representatives and sector-specific professionals (Figure 1). This mix provided the skills and knowledge needed for advisory duties and leading activities for community benefit.

In addition, the diverse mix of CAG members’ experiences created a dynamic within the group, where each member had areas of expertise and inexperience. The resulting combination of people made a CAG with diverse yet complementary skillsets enabling within group learning. Providing education opportunities, often through group training, to develop the skills of CAG members enables effective contribution for CAGs with novice members (Shippee et al., 2015). In this CAG, acknowledgement and openness to members’ inexperience and expertise allowed members to share experiences and knowledge and align their skills and interests with opportunities to upskill through participation in activities. Member development was achieved through interactive meetings, working groups for specific activities, and support for members to attend events or training (internal and external to the Program).

#### 6.2.3 Resources available to the CAG

CAGs are dependent on the generosity and commitment of the members that volunteer their time and skills. It is recognised that the remuneration of consumer and community volunteers is an integral part of supporting CCE participation (Gunatillake et al., 2020). However, volunteer remuneration is not the only type of resourcing to consider to achieve effective CAG participation in research and health services. For example, the number of activities the CAG led or engaged in would not have been possible without the Program’s staff allocation to support the CAG and funds to undertake activities. To support these requirements, implementers need to have budget and staffing incorporated into project plans, including further funding requests when projects are being developed. The Program’s provision of funds for CAG activities expanded the group’s scope, capacity, and, ultimately, impact. While community-led activities may not be the intent of all CAGs, if it is an expectation of implementers or CAGs, there needs to be proper support of the activities themselves that does not entirely rely on volunteers or the CAG to seek their own funding.

## 7 Conclusion

CAGs are one mechanism of incorporating CCE in health and medical research and health service delivery (McClean and Trigger, 2017; Stewart et al., 2019). The purpose, structure and responsibilities of CAGs vary depending on the expected contribution to a project or organisation. The way the Queensland Genomics CAG was structured and supported by the Program to function was key to its ability to operate in the dual roles of advisors to a health implementation program and leadership in community focus research and activities for the Program. Key enablers for these dual roles included; the diversity of CAG members’ skills and experience, resources provided to the CAG and the CAG’s ability to self-determine their direction. These factors proved essential to the functioning of the CAG with dual roles and ultimately to the impact of CAG activities in addressing unmet needs in the Program and sustaining engagement of the CAG members in the Program’s objectives.

## Data Availability

The original contributions presented in the study are included in the article/Supplementary Material, further inquiries can be directed to the corresponding authors.
