# Supplementary material for: Community input in a genomic health implementation program: Perspectives of a community advisory group
Source: Front Genet. 2022 Jul 22;13:892475. doi: 10.3389/fgene.2022.892475 (PMC9355292; doi:10.3389/fgene.2022.892475)
Supplement: Supplementary file 1 [file DataSheet2.PDF]

frontiers

Supplementary Material 2

Table S1: Considerations when forming a community advisory group as part of a research program.

Consideration for CAGs	Our CAG's experiences	Our Recommendations to improve CAG outcomes
Mechanisms for involvement	The Program desired CCE, but this was not embedded in the Program's projects' delivery requirements. CAG members actively advocate for more engagement with the Program's other projects. Without mechanisms in place, the CAG was limited in its ability to push from engagement.	- Establish requirements for CCE in projects through milestone or contract-based mechanisms.<br>- Stipulate the requirement for CCE in funding application process.<br>- Program needs to make projects aware of CCE support options that are available.
Education of researcher and health service personnel about CCE	Some project leads were reluctant to incorporate CCE or engage the CAG, as they felt their project did not need this type of input.	- Support staff training and education on CCE<br>- Provide opportunities for project leads to experience engaging with the community in research
Resourcing	Being provided financial and human resources was a critical enabler for running the CAG's advisory role and its activities.	- Budget for remuneration of CAG and meetings<br>- Allocation of staff time and duties to support CAG<br>- Fund CAG-led activities or support grant bids to undertake CAG-led activities if this is an identified priority<br>- When resources are not available to support activities create realistic expectations for workload based on member availability.
Professional structure	This CAG was run like a board, enabling ethical and professional conduct, which is necessary for how CAG is perceived when working with external stakeholders.	- Documentation of expectations. For example, terms of reference and strategic plan<br>- Outline member roles and responsibilities<br>- Conflict of Interest register and expectations for code of conduct<br>- Meeting agenda and minute taking, including task allocation and follow-up

Consideration for CAGs	Our CAG’s experiences	Our Recommendations to improve CAG outcomes
Group diversity and experience	The structuring of the CAG to have members with diverse experiences and perspectives was beneficial to providing advice and developing and executing community-led activities.	- Have a CAG recruitment strategy targeted to the individual project or program needs, including a plan for training (if needed)<br>- Ensure recruited people have the capacity to participate (i.e. time, skills, and informed) and are aware of requirements for participation in CAG<br>- Have people with a mix of experience levels to bring different perspectives.
Equity of participations	CAG membership usually is for a set term (1-2 years). This term provides more opportunities for the consumer to participate in the engagement process. Here membership was sustained for the Program's duration. This continuation balanced in regular CAG member change as this can reduce productivity. In cases where contributions are one-off or time-limited, regular changes can be facilitated, but this lack of continuity can be disruptive when contributing to long-term projects. Therefore, in developing a CAG, there needs to be a consideration of the proportion of short-term and long-term activities and how best to facilitate equity in participation.	- Consider intent for CAG and set terms of participation accordingly<br>- Provide opportunities for consumers and the community to participate in the Program outside of CAG.
Capability/ Readiness	The CAG developed during the first year. Therefore, CAG would not have been ready to take on projects seen later in the Program.	- Support the development of CAG capabilities as individuals and as a group. This will vary between groups, including training or upskilling members and the time needed for establishing the CAG.<br>- Plan for and undertake a review of the CAG

Consideration for CAGs	Our CAG's experiences	Our Recommendations to improve CAG outcomes
Expectation Management	The CAG was set up to advise on the Program's projects as a core function, but the optional nature of CCE and consultation with CAG for the Program's projects were not clearly communicated to members.	- Identify preconceptions and expectations of individuals and the group<br>- Communicate changes (internal and external)<br>- CAG members are volunteers, so be realistic about the milestones and outputs expectations of a CAG<br>- Create and communicate a plans for participation requirements
Workload	Dual role but predominantly worked on self-defined activities. Had there been a better linkage between CAG and Program projects or Program design, would CAG members have had the capacity to contribute? The level of burden and the subsequent issue of volunteer burn-out can be a considerable barrier to CAG engagement. Dual roles can create more work. The time commitment to CAG activities dominated duties in the latter half of the Program; there would not have been time for genuine engagement with the Program's projects.	- Assign roles rather than using the whole group for all activities (here, we used working groups)<br>- Supply adequate resources to cover workload – staffing support or financial or outsourcing contracts<br>- Prioritise tasks – volunteers cannot do everything<br>- Communication in advance of requirements to contribute so that members can plan availability.
Relationships	Working relationships were important for the CAGs ability to function effectively. They enabled; meaningful discussions in meetings, collaborative development of activity ideas, honest and constructive feedback, and the identification of gaps and external opportunities for the CAG to address.	- Provide opportunities for the CAG members to develop their relationships through face-to-face meetings and training opportunities.<br>- Have dedicated staff for working with CAG<br>- Have senior staff involved in CAG meetings (where appropriate)
