# Supplementary material for: Community input in a genomic health implementation program: Perspectives of a community advisory group
Source: Front Genet. 2022 Jul 22;13:892475. doi: 10.3389/fgene.2022.892475 (PMC9355292; doi:10.3389/fgene.2022.892475)
Supplement: Supplementary file 2 [file DataSheet1.PDF]

frontiers

# *Supplementary Materials 1*

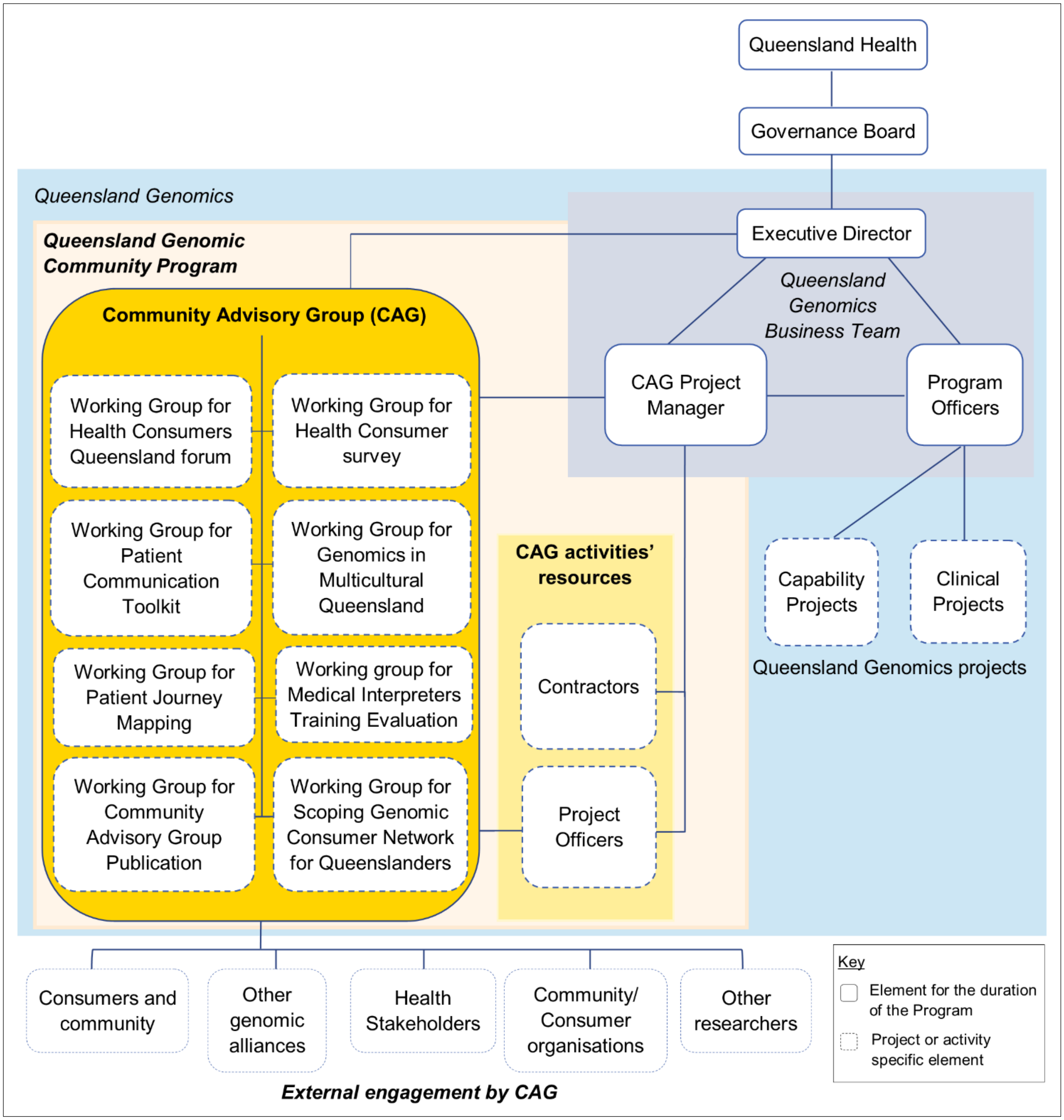


**Figure S1. Community Advisory Group interactions with Program, program governance and projects.**

Queensland Genomics

## Queensland Genomics Community Group

**The Queensland Genomics Community Group is an active collaborator within the health system working with researchers, clinicians and communities to facilitate equitable and accessible person-centred care for all Queenslanders.**

## Objectives

- To collaborate with Queensland Genomics and support its vision and mission
- To serve as an active conduit to consult, promote, and engage the people of Queensland in the work of Queensland Genomics and its partners
- To support the development of a health system that delivers the earliest possible diagnosis, appropriate intervention and follow up, and responds to patient needs in a flexible and nuanced person-centered way
- To advise Queensland Genomics projects on better ways to link and communicate with patients and their families, helping to ensure a patient and delivery focus

W queenslandgenomics.org

Queensland Genomics

## Themes of our work

### Collaboration

The Queensland Genomics Community Group supports and drives collaboration and communication between community, consumers, advocacy groups, clinicians, health systems, researchers, locally, nationally and internationally, and others, with the aim of informing the implementation of genomics into healthcare.

### Equity & Access

The Queensland Genomics Community Group supports equity of access to genomic medicine regardless of location, education, language, socio economic status, or health literacy. The Queensland Genomics Community Group works to improve clarity of treatment pathways, diagnostic tools and genomic medicine.

### Education

The Queensland Genomics Community Group builds education on different perspectives of genomic medicine within communities of health consumers, carers, clinicians, researchers, health administrators, advocates.

### Advocacy

The Queensland Genomics Community Group is a respected group who advocates for the community with government, NGOs, private sector to enable patient-centred personalised medicine now and into the future.

### Foundation & prioritisation

The Queensland Genomics Community Group recognises its role as laying down the tracks and setting the direction to build long term changes in the health system for the introduction of genomics. We work to help prioritise activities, balancing the desire to make a difference not only today, but also in the longer term.

### Real world context

The Queensland Genomics Community Group helps to frame actions and decisions within the real-world context experienced by consumers and community. We work to improve the understanding of researchers, clinicians, and decision makers in the health system about the real-world impacts of decisions, and the current experiences and challenges of consumers, families and communities.

## Areas of Action: our work with key stakeholders

### Queensland Genomics

- Collaborate with Queensland Genomics and support its vision and objectives
- Serve as a conduit to consult and promote, and to engage the people of Queensland in the work of Queensland Genomics and its partners
- Focus on consumer issues – in Queensland Genomics projects and the broader program
- Ensure activities of Queensland Genomics are relevant and beneficial to patients and communities across Queensland

### Community

- To be a voice for all Queenslanders
- Engage with the community – educate so that utility and limitations of genomics is understood, and community concerns addressed. Use positive stories regarding genomics to build awareness and understanding
- Increase awareness about the lives of people with genetic conditions. Help to bridge knowledge gaps
- Ensure the views of different sectors of the Queensland Genomics community are considered in implementing genomics

### Clinicians

- Enable clinical understanding in 'real world' context
- Support better education of clinicians in medical training, and ongoing professional development

### Health System

- Lay the foundations for health system to implement genomic medicine, ensuring diverse community voices, needs and issues are understood and responded to
- Support development of patient-centred policy, design and implementation
- Provide community input into patient-facing resources
- Provide a patient/community voice to enrich the application of genomics for patient benefit
- Create change and make genomics more accessible
- Help to identify future important areas in genomics where Queensland can have the greatest impact
- Help prioritise activities and balance what makes a difference today with what makes a difference in the longer term
- Provide a reality check about the consumer experience and the challenges of getting on, or being on a treatment pathway and the impacts that this has on the consumer and their family, friends and community

### Research

- Advise research projects on better ways to link and communicate with patients
- Support a community voice in development of genomics research and policy
- Ensure research focus remains on improved healthcare and patient needs

**Figure S2: Queensland Genomics Community Group's strategic plan.**
